# Lemierre Syndrome with Extensive Thrombosis: A Unique Case Report and Literature Review

**DOI:** 10.1155/2024/6335543

**Published:** 2024-09-03

**Authors:** Stergos Koukias, Asimenia Athousaki, Dionisios Klonaris, Melina Kavousanaki, Georgios Papazoglou, Nikolaos Papanikolaou

**Affiliations:** ^1^ 1^st^ Department of Internal Medicine General Hospital of Heraklion “Venizeleio-Pananeio”, Heraklion, Crete, Greece; ^2^ Department of Otorhinolaryngology-Head and Neck Surgery General Hospital of Heraklion “Venizeleio-Pananeio”, Heraklion, Crete, Greece

## Abstract

**Background:**

Lemierre syndrome (LS) is a rare complication of upper aerodigestive tract infections characterized by proximal and distal septic emboli, commonly including internal jugular vein (IJV) thrombosis. Diagnosis can be challenging, and treatment delays can result in increased patient morbidity and mortality. We present a rare case of LS with extensive thrombosis and multiple sites of distal infection and a narrative review of the literature. *Case Presentation*. A 52-year-old Caucasian male was transferred to the emergency department (ED) with an altered level of consciousness and clinical findings of acute bacterial pharyngotonsillitis. Medical history included cervical spine disorder and traumatic brain injury in the past, as well as the recent use of pain relievers due to acute cervical pain. Imaging studies revealed left IJV thrombosis that extended into multiple venous cerebral sinuses and infiltrates of the right lung. LS was considered the most likely diagnosis. The patient was intubated and transferred to the intensive care unit (ICU). Treatment included intravenous broad-spectrum antibiotics and anticoagulation therapy. Response to treatment was satisfactory. After extubation, he was transferred to a ward and discharged with resolution of clinical and imaging findings.

**Conclusion:**

LS is a rare disease and may have an insidious course. Timely diagnosis and appropriate treatment strategies, mainly broad-spectrum antibiotics, offer favorable outcomes in otherwise healthy individuals. The indications for anticoagulation therapy still remain controversial. Anticoagulants are usually administered to patients with extensive thrombosis. Surgical treatment includes abscess drainage, while IJV ligation and excision are reserved for nonresponders to medical treatment.

## 1. Introduction

Lemierre syndrome (LS) is a rare disease first described in 1936 by Andre Lemierre and typically refers to an oropharyngeal bacterial infection complicated by septic thrombophlebitis of the internal jugular vein (IJV) [1]. It occurs mainly in young male adults [2, 3]. The obligate Gram-negative anaerobic bacterium *Fusobacterium necrophorum* (FN), part of the oropharyngeal flora, constitutes the most common causative organism of this syndrome. However, in many cases, several species of *Streptococcus* also participate in the pathogenesis. Given the inherent resistance of FN to macrolides, quinolones, tetracyclines, and aminoglycosides and its ability to produce beta-lactamases, empirical antibiotic therapy should include combination therapy that targets FNs producing beta-lactamase and oral and pharyngeal streptococci [4–7]. Appropriate antimicrobial therapy is vital for a favorable disease outcome, considering the high mortality rate in the pre-antibiotic era and the subsequent significantly lower 5% rate in the last two decades [8, 9]. We present a rare case of LS with extensive thrombosis and multiple distal infection sites.

## 2. Case Presentation

**Figure 1:**
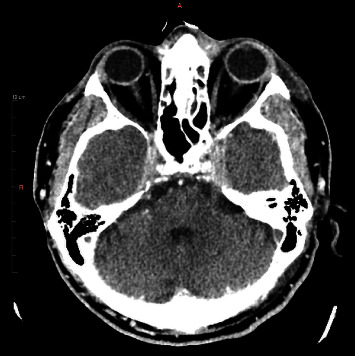
Contrast-enhanced brain CT showing thrombosis of multiple venous cerebral sinuses. The paranasal sinuses, as well as the middle ear and mastoid cavities, are free from fluid collection.

**Figure 2:**
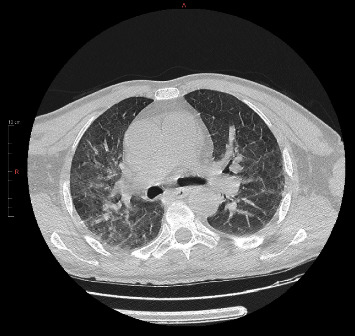
Chest CT showing ground-glass opacities and infiltrate of the right lung.

A 52-year-old male, with a medical history of cervical spine disorder and traumatic brain injury in the past, was transferred to the emergency department (ED) due to altered level of consciousness and dizziness. His relatives reported that during the last month, the patient suffered from severe intractable cervical pain and was treated with nonsteroidal anti-inflammatory drugs, tramadol, and physical therapy. The Glasgow Coma Scale was 8/15, so the patient was intubated. Empirical prompt antibiotic therapy with ceftriaxone and vancomycin was initiated due to high clinical suspicion of central nervous system (CNS) infection. Brain computerized tomography (CT) revealed extensive thrombosis of multiple venous cerebral sinuses (transverse, sigmoid, and cavernous sinuses bilaterally and superior sagittal sinus), as well as IJVs and retinal veins bilaterally (Figure 1). The findings of chest CT were ground-glass opacities of the right lung (Figure 2). The laboratory test results revealed marked leukocytosis and increased levels of C-reactive protein. A lumbar puncture was performed. Cerebrospinal fluid (CSF) analysis showed a total leukocyte count of 438 per liter and a differential neutrophil count of 85%. The CSF and blood cultures were sterile. Further physical examination revealed edema and fluid collection in the left temporomandibular joint and the left parotid gland. Paracentesis and aspiration of both fluid collections were performed. The fluid leukocyte count and differential were unremarkable, while the fluid culture was sterile.

The patient was intubated in the ED and transferred to the intensive care unit (ICU), where he remained for 10 days. He was treated with intravenous ceftazidime/avibactam, vancomycin, ampicillin, and metronidazole. He was also treated with enoxaparin for thrombosis. Post-extubation, the patient was transferred from the ICU to a general ward in the Department of Internal Medicine.

**Figure 3:**
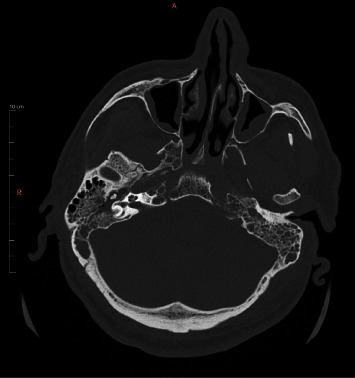
Brain CT showing fluid collection in both the middle ear and the mastoid cavities.

**Figure 4:**
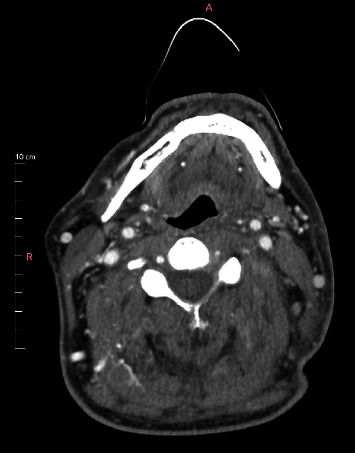
Contrast-enhanced neck CT showing persistence of the IJV thrombi, asymmetry of the left pharyngeal wall, and enlargement of the left carotid and post-styloid parapharyngeal spaces.

A new brain and neck CT revealed fluid collection in both the middle ear and mastoid cavities (Figure 3), as well as persistence of the IJV thrombi, asymmetry of the left pharyngeal wall, and inflammatory changes in the left carotid and post-styloid parapharyngeal spaces (Figure 4).

ENT head and neck examination revealed an asymmetry of the left lateral pharyngeal wall, indicative of inflammation of the ipsilateral pharyngeal band and deep neck spaces. Otomicroscopy revealed bilateral serous otitis media. Myringotomy was performed on both sides and serous otomastoiditis was diagnosed. However, this diagnosis would not explain intracranial complications on admission, since it was not present at the time. No significant enlarged lymph nodes or masses were found on head and neck examination. On flexible fiberoptic endoscopy, seropurulent secretions were identified in the nasal cavity, and uncomplicated acute bacterial rhinosinusitis was diagnosed. The asymmetry in the left lateral pharyngeal wall was confirmed, with a normal appearance of the supraglottic larynx.

Taking into account the medical history, the findings of the physical examination, and the medical imaging, LS was considered the most probable diagnosis. Empirical antibiotic treatment was modified to intravenous meropenem, linezolid, and metronidazole against common LS and other deep neck space and CNS infection bacteria, such as methicillin-resistant *Staphylococcus aureus* (MRSA), vancomycin-resistant *Staphylococcus aureus* (VRSA), and FS. Anticoagulation therapy was also modified to per os acenocoumarol.

Intravenous antibiotic treatment continued for three weeks. During this time, the patient became afebrile and hemodynamically stable. The laboratory test results returned to normal levels, and the blood cultures were sterile. He was discharged from the hospital on per os linezolid and metronidazole for six weeks, as well as per os acenocoumarol for six months. On discharge, contrast-enhanced head and neck CT revealed no change in the left transverse and sigmoid sinuses, while a significant reduction of thrombus extension was observed in the rest of the venous sinuses. The patient was scheduled for monthly outpatient medical visits. After six months, he completely recovered with no neurological deficits or abnormal findings on physical examination.

## 3. Discussion

According to the bibliography, LS is a rare disorder [7]. However, since the 1980s, there has been an upward trend in the number of reported cases [10]. The appearance of resistant microorganism strains due to overprescription of antibiotics for pharyngotonsillitis could be an explanation for this observation [11]. A large prospective study in Denmark reported that the overall incidence of LS is up to 3.6 cases per million and considerably higher (14.4 cases per million) in the age subgroup of young adults (14–25 years) [2]. The reason why young adults are more vulnerable to LS remains unclear. It has been suggested that the conversion from aerobic to anaerobic oral and pharyngeal flora in late childhood and puberty could be a possible explanation [12].

The most common primary site of infection in LS is the palatine tonsils, through which pathogens can invade the deep neck spaces. Subsequently, septic thrombophlebitis of the IJV, septic emboli, and infection of other distal sites can occur. In one published case report, pharyngitis was considered the primary site of infection with subsequent extension to the left parapharyngeal space, followed by metastatic infection of the lungs and the CNS. The most common site of metastatic infection in LS is the lungs, while the CNS is rarely implicated [13].

The release of proinflammatory cytokines at the site of infection could be the main culprit causing endothelial injury and venous thrombosis, typical features of LS [14]. Infection spreading through tissue, hematogenous, or lymphatic routes is considered the main mechanism of disease pathogenesis [7]. In our case report, the patient suffered from extension of infection into the IJVs, multiple venous cerebral sinuses, and the retinal veins.

A recent meta-analysis confirmed that the most common site of thrombosis and septic thrombophlebitis is the IJV and reported a wide spectrum of sites of thrombosis in LS, including the cerebral venous sinuses and the retinal veins [5]. The author also reported the results of the blood and tissue cultures of the patients. In 7.9% of the cases, the cultures were sterile and the responsible microorganism was not identified [5]. The main responsible organism is FN, which constitutes part of the normal oral and pharyngeal flora [5, 12]. Several other species of the same genus (e.g., *Fusobacterium nucleatum*), *Streptococcus* spp., *Peptostreptococcus* spp., *Staphylococcus* spp. (including MRSA), and *Bacteroides* spp. have been described as possible pathogens [5, 6, 10]. In our case report, no responsible microorganisms were identified. This can be explained by the inherently demanding and difficult cultivation of FN and anaerobes in general [15].

The administration of broad-spectrum antibiotics is the mainstay of empiric therapy for LS, pending the culture and susceptibility results. Antibiotic regimens should target FN and oral streptococci. The constitutive resistance of FN in macrolides, fluoroquinolones, tetracyclines, and aminoglycosides and beta-lactamase production by FN have been shown in many previous studies [6, 11, 13, 16, 17]. Antibiotics such as beta-lactams combined with a beta-lactamase inhibitor, metronidazole, clindamycin, and carbapenems appear to be efficient drugs, showing good in vitro susceptibility in isolated species of FN [18, 19]. The duration of therapy should be three to six weeks. Intravenous antibiotics can be switched to oral regimens as soon as infection is controlled [20]. In our case report, the patient was initially treated in the ICU with a variety of antibiotics (ceftazidime/avibactam, vancomycin, ampicillin, and metronidazole) against CNS infection and pneumonia. Although ampicillin administration is not recommended due to the high rates of antimicrobial resistance compared to piperacillin/tazobactam and carbapenems, *Listeria* spp. CNS infection was also in the differential diagnosis and had to be managed. Patients with proven CNS infection need broad-spectrum antibiotic coverage, penetrating the blood-brain barrier (e.g. vancomycin, third-generation cephalosporins, and metronidazole) [15]. When the diagnosis of LS was established after the patient was transferred from the ICU to a general ward, antibiotic treatment was modified to meropenem, linezolid, and metronidazole targeting MRSA, VRSA, and FN, considering sterile blood cultures.

Anticoagulation therapy for the management of septic embolism in LS remains contentious and is a subject of controversy between clinicians. Several studies showed clinical improvement regardless of anticoagulation therapy administration [21, 22]. Significant improvement was found in a retrospective study published by Rebelo et al. [23]. The impact of anticoagulation on vessel recanalization and mortality was not statistically significant in patients administered anticoagulation in a recent meta-analysis. In the bibliography, anticoagulation therapy has been proposed in high-risk patients when there are no contraindications. The rationale is that it accelerates thrombus lysis and prevents relapses, especially in intracranial thrombosis [24–26]. Low-molecular-weight heparin (LMWH) and warfarin have been widely used for the treatment of thrombosis in LS. In terms of children, exclusive administration of LMWH is recommended [22, 27]. The optimal duration of therapy is not yet clear. In our case report, anticoagulation was considered important due to extensive thrombosis of the left IJV and multiple cerebral venous sinuses. The duration of therapy for six months has been recommended. In patients with multiple sites of thrombosis, the administration of warfarin or acenocoumarol has been proposed with simultaneous INR monitoring, targeting 2.5 [28].

In the pre-antibiotic era, due to the high mortality rates of LS, surgical treatment was considered standard of care, given the paucity of therapeutic means. Ligation and excision of the thrombosed IJV remained the only viable option for the prevention of fatal outcomes [10]. The rationale behind this was to remove the site of sustained proliferation of pathogens inside blood clots and prevent their release to the circulation. Studies have also shown limited penetration of antibiotics into intravascular thrombi, leading to decreased response to treatment [24]. Currently, surgical treatment is aimed to abscess drainage, while surgical excision and ligation of the IJV are reserved in cases with a poor response to antibiotic therapy [27].

## 4. Conclusion

LS is a rare disease and, if untreated, has high morbidity and mortality rates. Due to its usually insidious course, a timely diagnosis and appropriate treatment strategies are imperative to favorable outcomes. Currently, the administration of broad-spectrum antibiotics is the mainstay of empiric therapy for LS. The indications for anticoagulation therapy still remain a matter of debate between clinicians, and such regimens are usually administered to patients with extensive thrombosis. Surgical treatment of LS includes abscess drainage, while IJV ligation and excision are reserved for nonresponders to medical treatment.

## Data Availability

All necessary data supporting the study findings are incorporated in the article.
